# Prenatal Diagnosis of Laterality Disorder Associated With NME7 Variants

**DOI:** 10.7759/cureus.119267

**Published:** 2026-11-07

**Authors:** Valentine Hardy, Matthieu Ortegat, Katrien De Clippel, Yves Sznajer, Wim Wuyts, Bettina Blaumeiser, Patricia Steenhaut

**Affiliations:** 1 Department of Obstetrics, Cliniques Universitaires Saint-Luc, Brussels, BEL; 2 Department of Obstetrics and Gynecology, Clinique Saint-Jean, Brussels, BEL; 3 Center for Human Genetics, Cliniques Universitaires Saint-Luc, Brussels, BEL; 4 Center of Medical Genetics, University and University Hospital Antwerp, Antwerp, BEL

**Keywords:** nme7, prenatal diagnosis, situs inversus, ultrasound, whole-exome sequencing

## Abstract

Situs inversus totalis (SIT) is a rare congenital laterality disorder characterized by a mirror-image reversal of the thoracic and abdominal organs. Although isolated SIT is generally associated with a favorable prognosis, prenatal diagnosis requires careful evaluation to exclude associated structural malformations and syndromic presentations, as well as genetic investigation to identify a possible underlying etiology and improve prognostic assessment. The genetic basis of SIT is complex and remains incompletely understood. To date, genotype-phenotype correlations for SIT-associated genetic variants remain poorly established.

We report the prenatal and postnatal clinical course of a fetus with isolated SIT carrying a homozygous intragenic deletion of the *NME7* gene, identified by whole-exome sequencing. A primigravida woman was referred at 22 + 2 weeks of gestation after dextrocardia was identified during the second-trimester morphology scan. Detailed ultrasound and fetal echocardiography were performed to assess organ laterality and exclude associated structural abnormalities. An invasive diagnostic procedure (amniocentesis) was performed, including chromosomal microarray analysis (CMA) and trio whole-exome sequencing. Ultrasound confirmed isolated SIT, characterized by dextrocardia, a right-sided stomach, and a left-sided liver and gallbladder. Echocardiography revealed normal cardiac anatomy and function. CMA identified a maternally inherited Xp22.33 deletion encompassing the entire *SHOX* gene, which was detected in the mother but not in the father and was considered an incidental finding. Trio whole-exome sequencing identified a homozygous, likely pathogenic intragenic deletion involving exons 2-7 of the *NME7* gene. Targeted parental DNA analysis confirmed that both parents were heterozygous carriers of the deletion. The pregnancy was continued based on the present molecular findings, since the implication of *NME7* variants seemed to be responsible for a non-syndromic form of SIT. A healthy female infant was delivered at term. Postnatal evaluation confirmed SIT with a small atrial septal defect with no hemodynamic impact and no other associated anomalies.

This case highlights the additional diagnostic value of whole-exome sequencing in fetuses with apparently isolated laterality abnormalities. It adds to the currently limited evidence linking *NME7* variants to isolated SIT. This case also emphasizes the importance of multidisciplinary assessment and genetic counseling to optimize diagnosis, prognostic evaluation, parental counseling, and postnatal management.

## Introduction

Abnormalities in lateralization, such as situs inversus totalis (SIT) and heterotaxy, are rare congenital anomalies characterized by abnormal left-right arrangement of the thoracic and abdominal organs, with a prevalence of one in 10,000 live births [1,2]. Although the diagnosis and management of lateralization abnormalities, particularly of the cardiovascular system, have improved in recent years, they still present challenges in the prenatal identification and postnatal management of associated cardiovascular and extracardiac malformations. SIT is a rare congenital malformation in which the abdominal and thoracic organs are arranged in a mirror-image configuration relative to their normal anatomical positions. Despite this abnormal arrangement, organ function is generally preserved [3].

This condition is caused by a defect in the specification of the left-right axis during early embryogenesis [2]. The etiology of lateralization abnormalities is complex and multifactorial, involving genetic and environmental factors [2]. Genetic analyses have identified variants in more than 80 genes involved in the establishment of left-right asymmetry and organ positioning [4]. Among these genes, *NME7* has been implicated in the development of laterality, with biallelic variants previously reported in association with situs inversus.

Published data on lateralization abnormalities have primarily focused on pediatric and adult populations. Studies of the genes involved have mainly focused on patients with primary ciliary dyskinesia (PCD) [5,6]. To date, little attention has been given to prenatal diagnosis, although precise prenatal assessment may allow more accurate prognostic evaluation and facilitate appropriate perinatal management. However, data correlating prenatal ultrasound findings with the identification of genetic variants in affected fetuses remain scarce [7].

## Case presentation

The patient is a Caucasian woman in her twenties with no medical or surgical history. This is her first pregnancy, which occurred spontaneously. The couple is not consanguineous. The patient was referred for a fetal medicine consultation after “situs inversus” was detected during a second-trimester ultrasound. Up to that point, her prenatal care had been uneventful. Her first-trimester ultrasound was normal, and she underwent non-invasive prenatal testing (NIPT), which showed a low risk for major chromosomal abnormalities and confirmed a female fetus (46,XX).

At 22 weeks and two days of gestation, ultrasound performed at our tertiary fetal medicine referral center confirmed isolated SIT, with the stomach and heart located on the right side, and the liver, gallbladder, spleen, and great vessels arranged in a mirror-image configuration. The fetus was appropriately grown for gestational age, the volume of amniotic fluid was normal, and fetal and uterine artery Doppler studies were normal. An evaluation by the fetal cardiology team confirmed dextrocardia with normal cardiac anatomy and function (Figures 1, 2).

**Figure 1:**
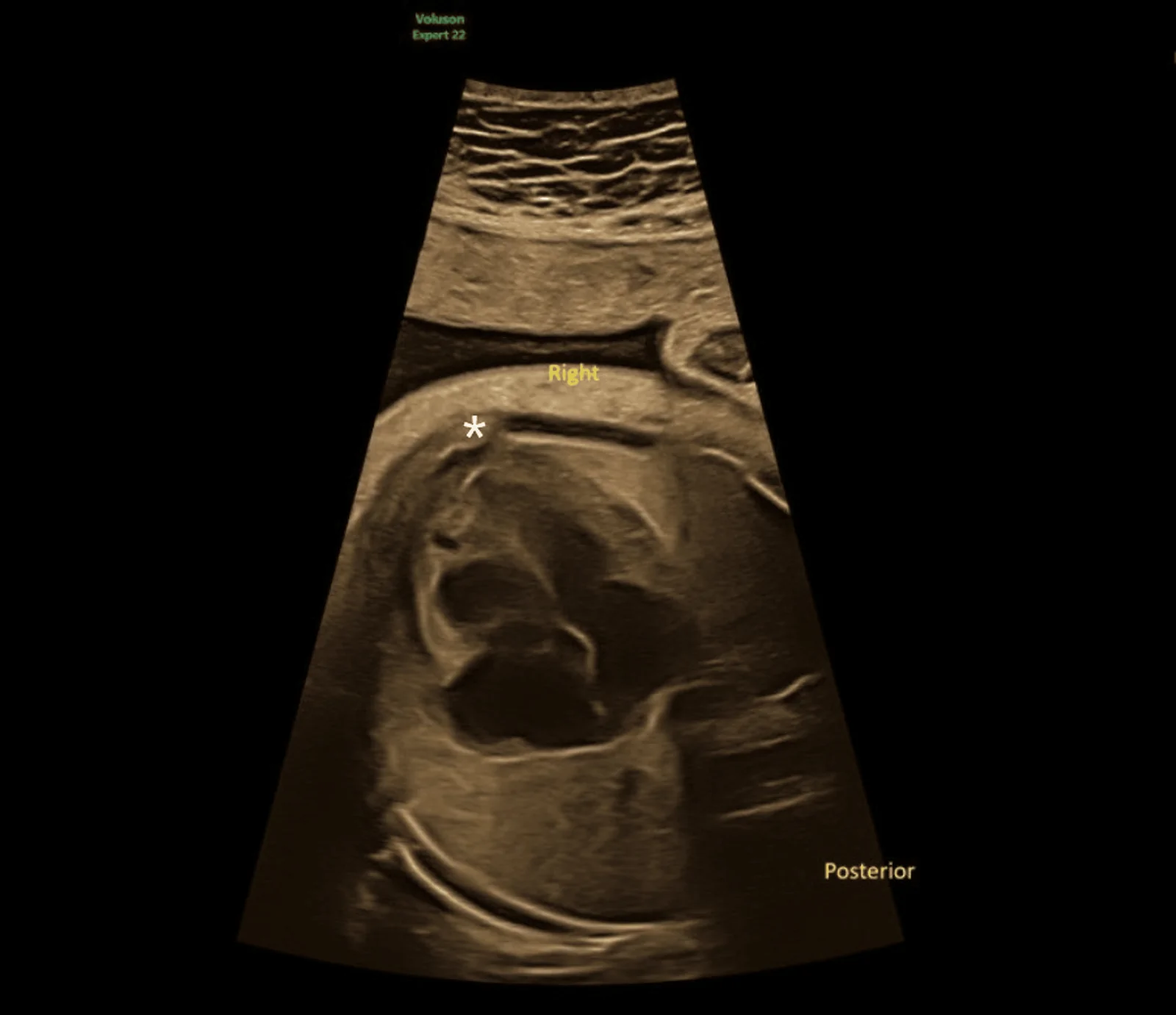
Prenatal ultrasound examination performed at 30 + 4 weeks of gestation Four-chamber view demonstrating dextrocardia, with the cardiac apex (*) pointing to the right.

**Figure 2:**
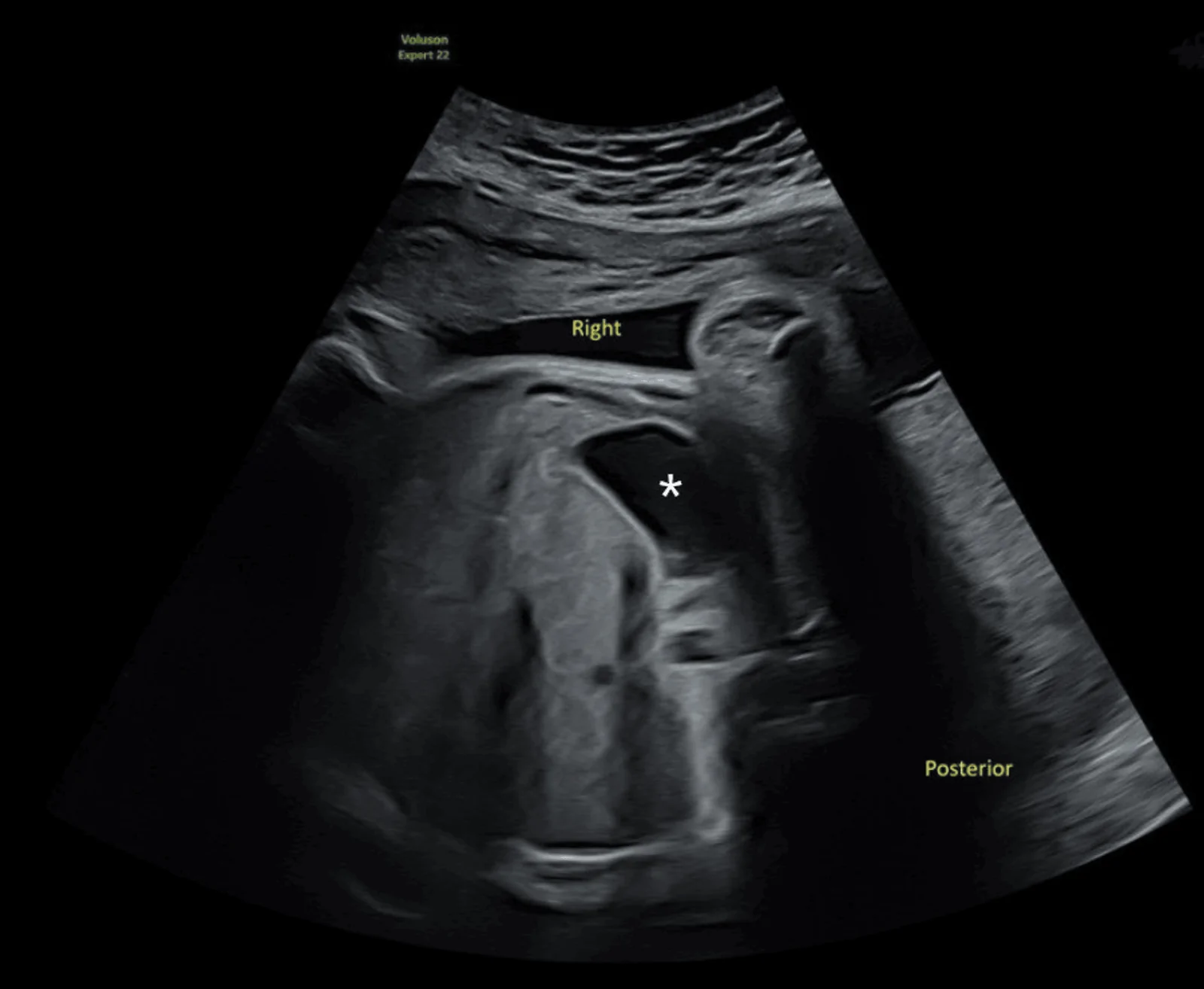
Prenatal ultrasound examination performed at 30 + 4 weeks of gestation Transverse abdominal view showing a right-sided stomach (*), consistent with situs inversus totalis.

Amniocentesis was performed at 23 weeks and two days of gestation to conduct genetic testing (Figure 3). Chromosomal microarray analysis (CMA) revealed an interstitial deletion at Xp22.33 encompassing the entire *SHOX* gene and its regulatory region. This deletion was of maternal origin and was considered an additional genetic finding, unrelated to the SIT, since haploinsufficiency of the *SHOX* gene is associated with Léri-Weill dyschondrosteosis and short stature but has not been associated with SIT [8,9]. The mother was of short stature. This finding was discussed during genetic counseling, including its potential clinical implications and the need for appropriate follow-up.

**Figure 3:**
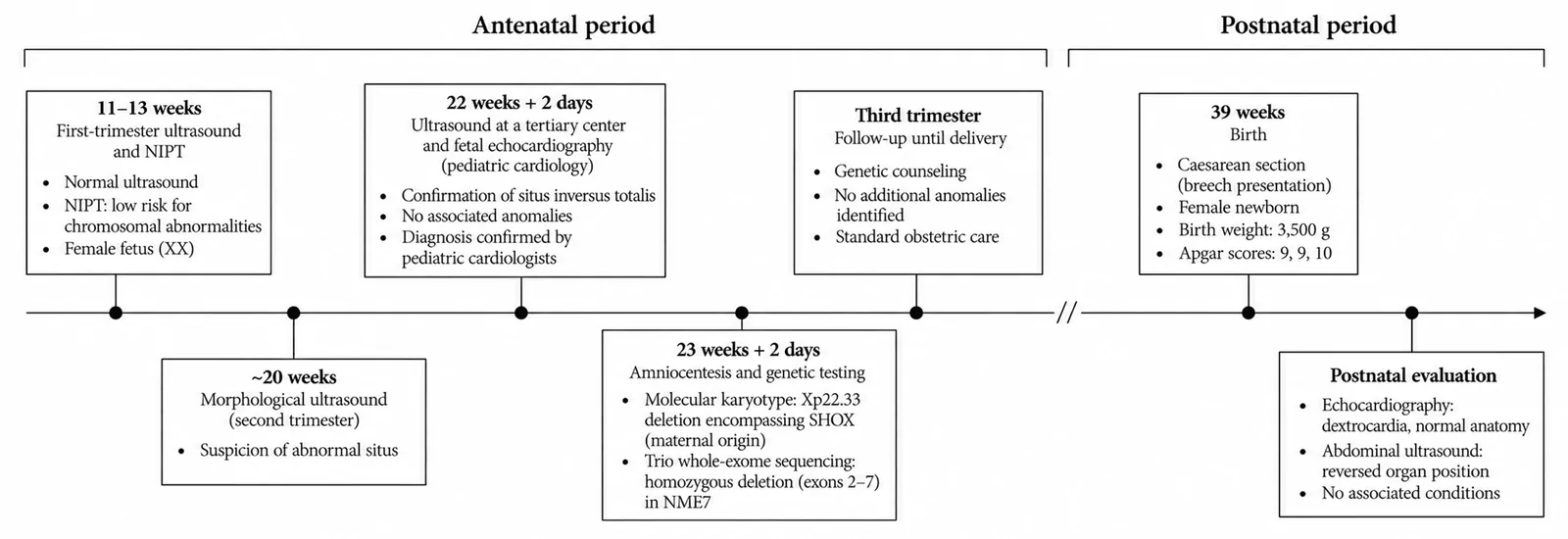
Diagnostic approach in our case of situs inversus totalis, including prenatal ultrasound assessment, amniocentesis, chromosomal microarray analysis, trio whole-exome sequencing, and postnatal evaluation NIPT: non-invasive prenatal testing

Given the genetic heterogeneity of SIT and the aim of further investigating its potential molecular etiology, trio whole-exome sequencing was performed. Fetal sequencing did not identify pathogenic variants in genes associated with PCD but revealed a homozygous intragenic deletion involving at least exons 2-7 of the *NME7* gene. Parental analysis showed that both parents were heterozygous carriers of the same deletion, consistent with an autosomal recessive inheritance pattern. The exact breakpoints of the deletion could not be determined by the available analysis. In the context of the reported association between *NME7* variants and situs inversus, this finding was considered the most likely molecular explanation for the isolated SIT [10].

Genetic counseling was offered, and the pregnancy was managed with standard obstetric care (Figure 3). The patient gave birth at 39 weeks via scheduled Cesarean section due to a breech presentation. The parents declined the external version and vaginal delivery of a breech fetus. The baby girl weighed 3,500 grams at birth. Her Apgar scores were 9, 9, and 10, one, five, and ten minutes after birth, respectively. No specific neonatal care was required. The newborn had a normal stay in the maternity ward. An abdominal ultrasound revealed that the abdominal organs, which are normally asymmetrical, were arranged in a mirror-image configuration. The aorta was located on the right side of the spine, and the inferior vena cava on the left. Whereas the liver and gallbladder appeared normal, they were located on the left. The spleen was located on the right.

Postnatal cardiac evaluation confirmed the diagnosis of SIT and identified a small atrial septal defect on echocardiography, without hemodynamic significance. No other associated cardiac or extracardiac abnormalities were detected. The newborn was discharged from the maternity ward on the fourth day of life and was scheduled for cardiologic follow-up because of the atrial septal defect and endocrinologic follow-up because of the *SHOX* gene deletion.

## Discussion

SIT is a rare anomaly of embryonic lateralization characterized by a mirror-image reversal/arrangement of the thoracic and abdominal organs [2]. Prenatal diagnosis primarily relies on a thorough analysis of fetal laterality during the second-trimester morphological examination [11]. When SIT occurs in isolated form, it is generally associated with a favorable prognosis. Thorough morphological evaluation is required for SIT diagnosis, particularly of the heart, to rule out heterotaxy or associated malformations that could alter management and neonatal prognosis [2]. Abnormalities in lateralization, including situs inversus and heterotaxy, are recognized as manifestations of certain ciliopathies, particularly PCD [12]. The latter is a genetically and phenotypically heterogeneous condition that significantly impacts quality of life due to chronic symptoms and progressive lung function deterioration [11,13]. PCD is characterized by chronic suppurative involvement of the sinuses, ears, and lungs; neonatal respiratory distress; lateralization abnormalities of asymmetric organs; and fertility problems [12,13]. It is a rare, clinically and genetically heterogeneous group of disorders characterized by dysfunction of motile cilia, with more than 50 genes identified to date [13].

Therefore, trio whole-exome sequencing was performed to further investigate the genetic etiology of SIT and identify a potential molecular cause in our case. No pathogenic variants were identified in genes associated with PCD. However, a homozygous intragenic deletion involving at least exons 2-7 of the *NME7* gene was identified. No other morphological abnormalities or neonatal complications were detected prenatally [10].

Sedova et al. [14] demonstrated that inactivation of the *NME7* gene in rats results in symptoms consistent with PCD. *NME7* is an established member of the ciliome and contributes to the regulation of the microtubule-organizing center. Homozygous deletion of this gene appears to result in a poor prognosis, as the majority of animals die before weaning due to neonatal complications. The most prominent phenotypes among survivors include hydrocephalus, SIT, postnatal growth retardation, and infertility in both sexes [14].

In the original human publication by Reish et al. [10], a homozygous *NME7* variant was associated with a non-syndromic SIT phenotype. The variant identified in the present case differs from that reported by Reish et al., as it consists of a homozygous intragenic deletion involving at least exons 2-7, whereas the previously reported variant was a splice-site variant affecting exon 10. In the present fetus, no associated morphological abnormality was identified, and the newborn adapted normally. Reported human *NME7* variants do not appear to consistently cause neurologic, cognitive, and/or extracardiac malformations. This suggests that the phenotype may be limited to an isolated laterality abnormality [10]. Nevertheless, the scarcity of published cases and limited clinical experience warrant caution before a definitively favorable prognosis can be established. Given the PCD-like phenotype observed in the *NME7* rat model and the limited number of reported human cases, the child will be clinically monitored for respiratory symptoms. Further evaluation for PCD will be considered only if respiratory symptoms suggestive of PCD develop during childhood. Our findings are consistent with previous reports of prenatal SIT and highlight the potential contribution of whole-exome sequencing to improving diagnostic accuracy [1]. Xue et al. [15] demonstrated that exome sequencing following a normal karyotype may optimize the detection of genetic variants associated with fetal dextrocardia, improve prognostic assessment, and guide neonatal care.

These data confirm the etiology, help refine genetic counseling and postnatal monitoring, and extend our understanding of the involved molecular mechanism. The concurrent identification of an unrelated maternal *SHOX* gene deletion may underscore the complexity of interpreting successive genetic work-up results and underline the prevalence of incidental findings [1]. This observation underscores the importance of a clinical genetics team that can provide support, ensure accurate interpretation of results, and appropriately provide patient information [15]. A detailed summary for the comparison of heterotaxy and SIT is mentioned in Table 1.

**Table 1:** Comparison of heterotaxy and situs inversus totalis [16-18]

Feature	Heterotaxy (situs ambiguus)	Situs inversus totalis
Definition	Abnormal and non-systematic arrangement of the thoracic and abdominal organs resulting from impaired left–right axis determination.	Complete mirror-image arrangement of the thoracic and abdominal organs, with preservation of normal anatomical relationships.
Embryological basis	Abnormal left–right axis determination involving laterality signalling pathways, including ciliary dysfunction.	Complete reversal of the left–right axis during embryogenesis without disruption of visceral organisation.
Visceral arrangement	Variable and discordant arrangement of the thoracic and abdominal organs (situs ambiguus).	Complete and concordant mirror-image arrangement of the thoracic and abdominal organs.
Cardiac position	Variable: levocardia, dextrocardia, or mesocardia.	Dextrocardia in most cases.
Congenital heart disease	Very common and frequently complex, including complete atrioventricular septal defect, univentricular heart, total anomalous pulmonary venous connection, transposition of the great arteries, and conotruncal abnormalities.	Uncommon; intracardiac anatomy is generally normal despite dextrocardia.
Splenic abnormalities	Asplenia, polysplenia, or abnormal splenic morphology, depending on the pattern of isomerism.	Morphologically normal spleen located on the right side.
Other associated abnormalities	Intestinal malrotation, interruption of the inferior vena cava with azygos continuation, abnormal systemic or pulmonary venous connections, and biliary or pancreatic abnormalities.	Usually absent; possible association with primary ciliary dyskinesia, including Kartagener syndrome.
Prognosis	Mainly determined by the severity of associated congenital heart disease and extracardiac abnormalities.	Generally favourable in the absence of associated cardiac abnormalities or syndromic conditions.

## Conclusions

This case report emphasizes the importance of a multidisciplinary approach that combines specialized prenatal ultrasonography, fetal cardiology expertise, and advanced genetic testing for evaluating lateralization abnormalities. It also expands the available data on *NME7* gene variants and emphasizes the need for further research to better characterize their phenotypic expression and prognostic impact during the prenatal period.
